# Neoadjuvant chemoradiation therapy for resectable esophago-gastric adenocarcinoma: a meta-analysis of randomized clinical trials

**DOI:** 10.1186/s12885-015-1341-7

**Published:** 2015-04-28

**Authors:** Tao Fu, Zhao-De Bu, Zi-Yu Li, Lian-Hai Zhang, Xiao-Jiang Wu, Ai-Wen Wu, Fei Shan, Xin Ji, Qiu-Shi Dong, Jia-Fu Ji

**Affiliations:** Department of gastrointestinal surgery, Peking University Cancer Hospital & Institute, Haidian District Fuchengmen Road No. 52, Beijing, 100142 China

**Keywords:** Preoperative chemoradiation therapy, Esophago-gastric adenocarcinoma, Overall survival, Meta-analysis

## Abstract

**Background:**

The efficacy and safety of preoperative chemoradiation therapy (CRT) for advanced esophago-gastric adenocarcinoma are still in question, and the prognosis of these patients is poor.

**Methods:**

We systematically searched electronic databases from January 1990 to July 2014. The primary outcome was overall survival. The secondary outcomes were a R0 resection rate, positive rate of lymph node metastasis, postoperative recurrence rate, pathological complete response (pCR) rate and perioperative mortality. Overall survival was measured with a hazard ratio (HR), while other secondary outcomes were measured with an odds ratio (OR).

**Results:**

Seven randomized controlled trials (RCTs) including 1085 patients were searched and, of these, 869 had adenocarcinoma. Patients receiving preoperative CRT had a longer overall survival (HR 0.74; 95% confidence interval (CI) 0.63–0.88), higher likelihood of R0 resection and greater chance of pCR, while they had a lower likelihood of lymph node metastasis and postoperative recurrence. The difference of perioperative mortality was non-significant. In addition, the result of the comparison between preoperative CRT and preoperative chemotherapy (CT) in two RCTs was non-significant.

**Conclusion:**

Patients with resectable esophago-gastric adenocarcinoma can gain a survival advantage from preoperative CRT. However, limited to the number of RCTs, the effect of adding radiotherapy to preoperative CT separately is still uncertain and more high-quality prospective trials are needed.

## Background

Throughout the world, adenocarcinoma of the esophagus, gastroesophageal junction and stomach rank among the most common cancers [1-3]. Additionally, during the past decade, there has been a dramatic increase in the incidence of gastro-esophageal junction cancer [4]. Adenocarcinoma accounts for a great majority of the cases of gastro-esophageal junction carcinoma in East Asia [5,6]. Furthermore, the prognosis of patients with these types of cancer is bleak [7,8]. Generally, surgery is the primary intervention for local advanced gastro-esophageal adenocarcinoma. However, the overall survival rates with surgery alone remain low, while the recurrence rates remain stubbornly high in most series [9]. The poor survival rates provide a strong rationale for the design of new treatment modalities.

As early as 1896, X-ray was first used in tumor therapy by Despeignes [10]. More than a century later, our understanding and development of radiotherapy led to a significant role in the comprehensive treatment of gastro-esophageal cancer. As patients can benefit from radiotherapy on a local control ratio, clinical experts can apply preoperative tumor down-staging and improve the resection rates of carcinoma. In addition, compared with postoperative radiotherapy, preoperative therapy is more accurate for the localization of the tumor [11]. However, as it is recognized as a systemic disease, patients with gastro-esophageal carcinoma should undergo chemotherapy as early as possible. If chemotherapy precedes preoperative radiotherapy alone, considering the interval between radiotherapy and surgery and the possible complications after surgery, the initial time of systemic chemotherapy will be further delayed. According to the sensitization of chemotherapy [12], Several phase II studies and RCTs have found that preoperative CRT has preferable safety and efficacy for local advanced gastro-esophageal adenocarcinoma [11,13-18].

Although some RCTs have proven the effectiveness of neoadjuvant chemoradiation therapy, there is the concern that meta-analysis would provide more powerful evidence for clinical decision-making relative to RCTs. However, the latest meta-analysis regarding preoperative CRT for gastro-esophageal carcinoma was published on 2007 [19], and the article only contains 3 RCTs that range from 1989 to 2006, while there were 4 new RCTs published from 2007 to 2014. Furthermore, the previous meta-analyses mainly focused on all types of gastro-esophageal carcinoma and contained not only preoperative CRTs but also preoperative chemotherapy [20,21], while this article focuses solely on adenocarcinoma and preoperative CRTs.

## Methods

### Literature search

To identify useful studies and published abstracts,we systematically searched electronic databases including the Cochrane Central Register of Controlled Trials (CENTRAL), PubMed, Excerpta Medica Database (EMBASE), the Cochrane Database of Systematic Reviews and the China National Knowledge Infrastructure (CNKI). There were no language restrictions. The medical subject headings were listed as follows: esophagus/gastroesophageal /gastric adenocarcinoma, preoperative chemoradiation therapy, and randomized controlled trials (RCT). The search included literature published from January 1990 to July 2014. We also reviewed all abstracts that were potentially relevant to our subject. Furthermore, other grey literature as well as unpublished work, ongoing studies and negative results were searched as well. Two investigators conducted the search independently, and their results were combined.

### Study review and inclusion

Two authors independently reviewed the study. The titles and abstracts were in agreement with the articles to be retrieved. To identify studies for the analysis, the inclusion criteria were designed as follows: (1) published RCTs that had a clear statement in the Materials and Methods section. (2) RCTs comparing preoperative CRT plus surgery with surgery alone or preoperative CRT plus surgery with preoperative chemotherapy plus surgery. (3) RCTs including patients with resectable, histologically proven adenocarcinoma of the esophagus, stomach or gastroesophageal junction without metastatic disease. (4) RCTs with a low risk of selection bias, performance bias, detection bias, attrition bias, reporting bias and other bias. Bias was assessed using Begg’s and Egger’s tests [22,23]. (5) Patient survival was used as the measureable outcome.

### Outcome measures

The primary outcome was overall survival, mostly based on an intention-to-treat analysis. The secondary outcomes were the R0 resection rate, which was defined by a tumor-free resection margin; positive rate of lymph node metastasis; postoperative recurrence rate; complete pathological response rate; and perioperative mortality.

### Statistical analyses

Data analysis was performed using Review Manager 5.2.0 for Windows. Overall, survival was measured with a hazard ratio (HR), while the R0 resection rate, positive rate of lymph node metastasis, postoperative recurrence rate and perioperative mortality were measured using odds ratios (OR). Furthermore, intention-to-treat (ITT) analyses were conducted when possible. If permitted, HR and the corresponding standard errors were obtained directly from the article; otherwise, they were calculated using the methods of Parmar [24], Tierney [25], and Williamson [26]. These approaches use confidence intervals, log-rank p-values, number of events and Kaplan–Meier survival curves to estimate the HR and standard errors. Moreover, the measures of HR and OR were investigated for statistical heterogeneity by I^2^ statistics, with a value of I^2^ > 50% indicating substantial heterogeneity. Where there was evidence of heterogeneity, subgroup analysis or sensitivity analysis were performed to investigate possible bias and derived summary estimates according to the random effect model; otherwise, the Mantel-Haenszel fixed effect model was used to compute the results. All of the significance tests were two-sided, with p = 0.05 as the cutoff.

## Results

### Identification of studies and features of the RCTs

**Figure 1:**
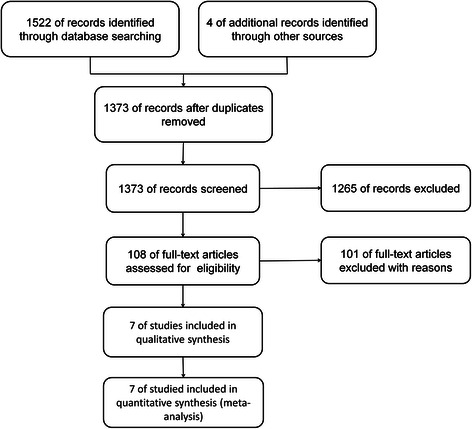
Preferred Reporting Items for Systematic Reviews and Meta-Analyses (PRISMA) diagram. The figure displays the information retrieval process for valuable articles and indicates the exclusion process of irrelative articles for this research.

**Figure 2:**
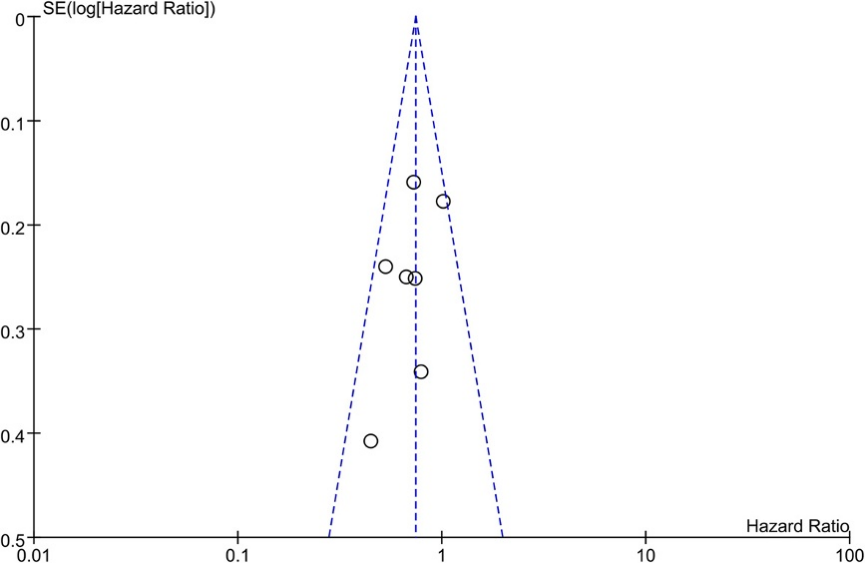
Funnel plots for the primary outcome. The horizontal axis corresponds to the study-specific HR which means the efficacy of the therapy. The vertical axis corresponds to the study-specific SE which means the size of the study. The circles represent the study included. The area of the dash line represents the range without bias in the study.

The results of the literature search are displayed in a Preferred Reporting Items for Systematic Reviews and Meta-Analyses (PRISMA) diagram (Figure 1). A total of 1522 studies were retrieved in the database, and 4 additional studies were found from other channels, such as conference reports, and so on. Among them, 1519 records were mostly unrelated to our subject, and only 7 RCTs met our inclusion criteria, which examined a total of 1085 patients. The main features of the trials included in the meta-analysis are shown in Table 1. The seven RCTs included 869 patients with esophago-gastric adenocarcinoma, 430 of whom received CRT before surgery. Approximately 5 RCTs focused on the topic of preoperative chemoradiation therapy (CRT) followed by surgery versus surgery alone [27-31], while 2 RCTs focused on the topic of CRT followed by surgery versus preoperative chemotherapy (CT) followed by surgery [32,33]. The cancer positions involved in the 6 RCTs were of the esophagus and gastro-esophageal junction, and 2 RCTs referred to the cardia. In addition, the median age of patients ranged from 56 to 65 year, and the proportion of females was 18.4%. Table 2 and Table 3 display some other characteristics regarding the RCTs included in our study. The total sample size of our meta-analysis was 1085, which contained 869 patients with adenocarcinoma, while the number of each RCTs varied greatly. The 3 year and 5 year overall survival (OS) rate are also displayed in Tables 2 and 3, where there was a certain difference between the CRT plus surgery group and the surgery alone or CT plus surgery groups. The treatment schedule is also listed (Tables 2 and 3). Additionally, no publication bias was found from the funnel plots (Figure 2).

**Table 1:** **Basic characteristics of the randomized controlled trials included**

Study and year	Country	Cancer position	R0 resection	Down-staging pCR of CRT	Journal publication
Walsh, [27]	Ireland	Stomach	NM	13(25.0%)	NEJM
		Esophagus			
Urba, [30]	USA	Stomach	90(92.8%)	9(24.3%)	JCO
		Esophagus			
TROG, [31]	Australia	Esophagus	179(69.9%)	NM	Lancet Oncol
CALGB9781, [28]	USA	Stomach	NM	10(40.0%)	JCO
		Esophagus			
Stahl, [33]	German	Stomach	84(70.6%)	7(11.7%)	JCO
Burmeister, [32]	Australia	Stomach	62(82.7%)	5(12.8%)	EJC
		Esophagus			
CROSS, [29]	Netherlands	Stomach	259(70.8%)	28(23.1%)	NEJM
		Esophagus			

*Note:* pCR: pathological complete response; CRT: preoperative chemoradiation therapy; CT: preoperative chemotherapy; NM: not mentioned.

**Table 2:** **Preoperative CRT versus surgery alone**

Study and year	Sample size	Treatment approach	Treatment schedule (CRT)	3 y or 5 y OS (CRT V. Surg)
Walsh, [27]	113	CRT-Surgery V. Surgery	40Gy/15f/15d	3y: 32% V. 6%
			5-Fu (15 mg/kg/d)	
				5y: NM
			Cisplatin (75 mg /m2)	
Urba, [30]	100	CRT-Surgery V. Surgery	45Gy/15f/15d	3y: 30% V. 16%
			Cisplatin (20 mg/m2/d)	
				5y: NM
			5-Fu (300 mg/m2/d)	
TROG, [31]	256	CRT-Surgery V. Surgery	35Gy/15f/3w	3y: 25.6% V. 24.1%
			Cisplatin (80 mg/m2)	
				5y: 11.5% V. 9.6%
			5-Fu (800 mg/m2)	
CALGB9781, [28]	56	CRT-Surgery V. Surgery	50.4Gy/28f/28d	3y: NM
			Cisplatin (100 mg/m2)	
				5y: 39% V. 16%
			5-Fu (1000 mg/m2/d)	
CROSS, [29]	366	CRT-Surgery V. Surgery	41.4Gy/23f 5f/w	3y: 39.6% V. 35.5%
			Carboplatin (2 mg/ml/min)	
				5y: 13.4% V. 7.1%
			Paclitaxel (50 mg/m2)	

*Note:* OS: overall survival; CRT: preoperative chemoradiation therapy; CT: preoperative chemotherapy; V: versus; Surg: surgery; 5-Fu: fluorouracil; NM: not mentioned.

**Table 3:** **Preoperative CRT versus preoperative CT**

Study and year	Sample size	Treatment approach	Treatment schedule	3 y or 5 y OS (CRT V. CT + S)
Stahl, [33]	119	CRT-Surgery V. CT-Surgery	**Scheme of CRT:** GTV30Gy/15f cisplatin (50 mg/m2), etoposide (80 mg/m2)	3y: 52% V. 49%
			**Scheme of CT:** 5-Fu (2 g/m2) leucovorin (500 mg/m2) cisplatin (50 mg/m2)	5y: 45% V. 36%
Burmeister, [32]	75	CRT-Surgery V. CT-Surgery	**Scheme of CRT:** Cisplatin (80 mg/m2) 5-Fu (1000 mg/m2/d) GTV35Gy/15f	3y: 47.4% V. 27.7%
				5y: NM
			**Scheme of CT:** Cisplatin(80 mg/m2) 5-Fu (1000 mg/m2/d)	

*Note:* OS: overall survival; CRT: preoperative chemoradiation therapy; CT: preoperative chemotherapy; V: versus; Surg: surgery; 5-Fu: fluorouracil; NM: not mentioned.

### Primary outcome

**Figure 3:**
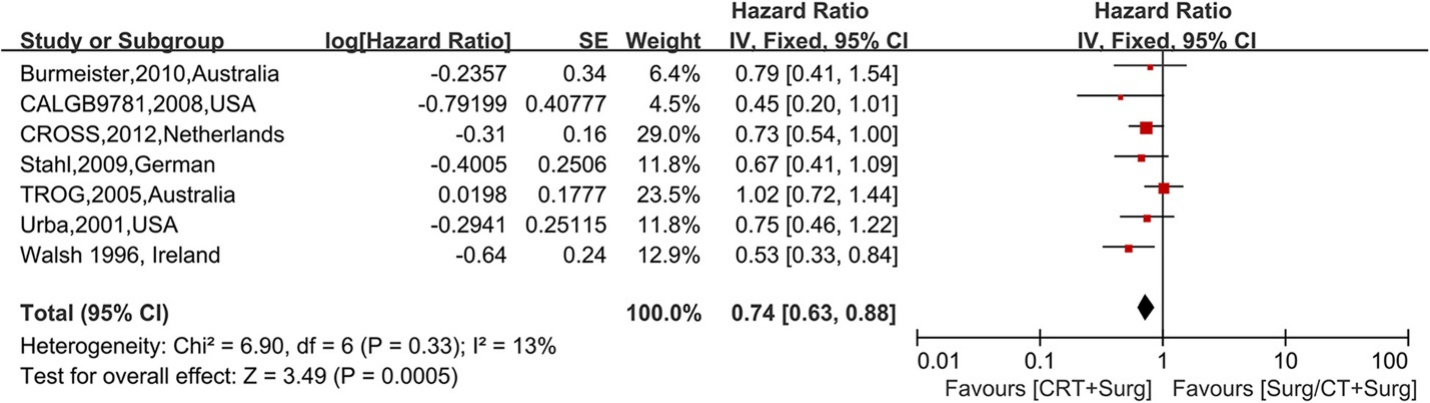
Forest plots for the primary outcome overall survival. The squares and horizontal lines correspond to the study-specific HR and 95% CIs. The area of the squares reflects the study-specific weight. The diamond represents the pooled HR and 95% CI.

The primary outcome examined in our study was overall survival and was reported in all seven RCTs. The meta-analysis gave the result that the pooled HR was 0.74 (95% CI 0.63–0.88) for the preoperative CRT plus surgery group compared to the preoperative CT plus surgery or surgery alone groups (Figure 3). It is noteworthy that four RCTs not only contained adenocarcinoma but also contained squamous cell carcinoma (SCC) [28-31]. Therefore, the individual HR of the CROSS trial and the TROG trial, excluding SCC, were calculated separately from the data given by the original article, and the results were 0.73 (95% CI 0.54-1.00) in the CROSS trial and 1.02 (95% CI 0.72-1.44) in the TROG trial. In the CALGB9781 trial and Urba’s trial, the individual data of SCC were not displayed; however, the number of SCC in these two studies was only 37. Compared to the total number of 869, the interference of these 37 SCC patients could be ignored. As the heterogeneity test was not statistically significant (I^2^ = 13%), the fixed effect model was used to calculate the result for OS. Figure 3 shows Forest plots for OS. The individual HR ranged from 0.45 (95% CI 0.20-1.01) for the CALGB9781 trial to 1.02 (95% CI 0.72-1.44) for the TROG trial; only a single individual HR favored the preoperative CRT group. This result together with those of the pooled HR indicated that there was a survival advantage for patients with preoperative CRT followed by surgery. In addition, according to the results of the heterogeneity test, there was no need to perform sensitivity analysis or subgroup analysis for the primary outcome.

### Secondary outcomes

**Figure 4:**
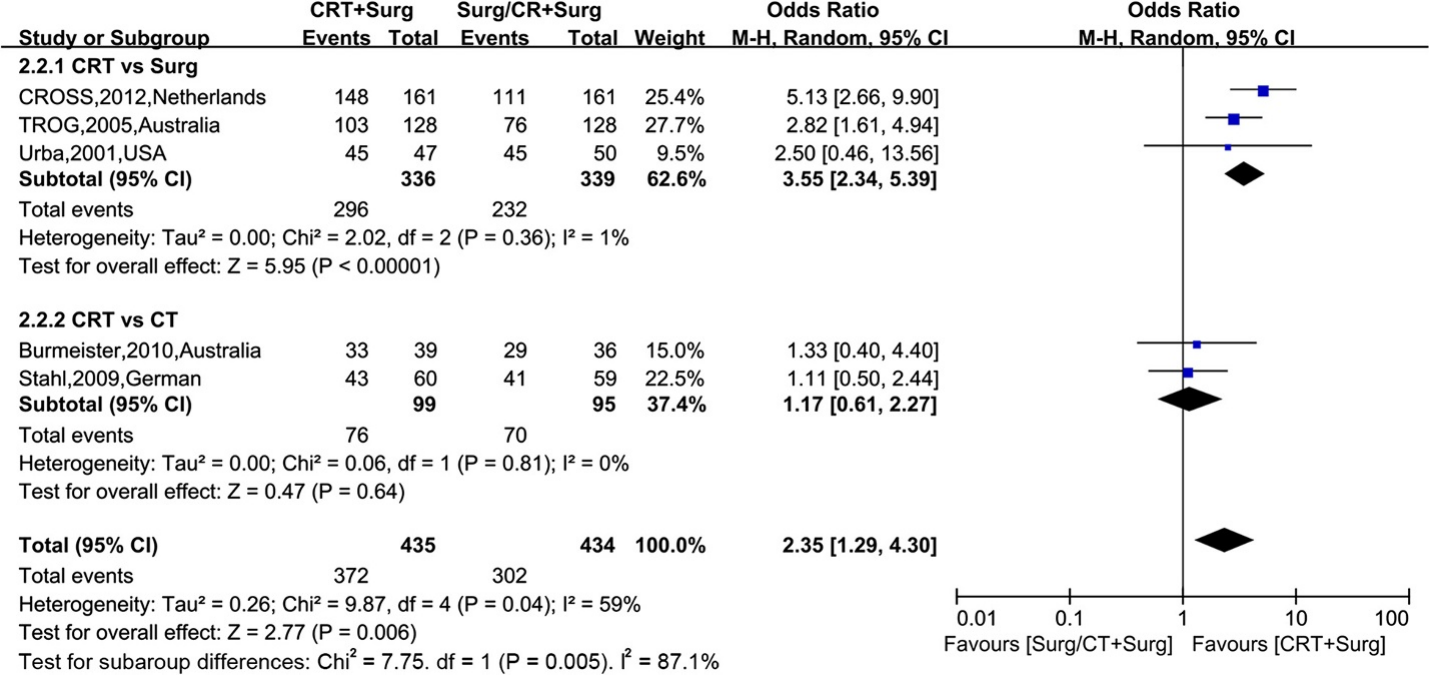
Forest plots for the secondary outcome the R0 resection rate by different control groups. The squares and horizontal lines correspond to the study-specific HR and 95% CIs. The area of the squares reflects the study-specific weight. The diamond represents the pooled HR and 95% CI.

**Figure 5:**
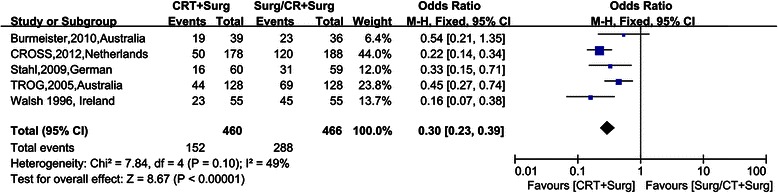
Forest plots for the secondary outcome positive rate of lymph node metastasis. The squares and horizontal lines correspond to the study-specific HR and 95% CIs. The area of the squares reflects the study-specific weight. The diamond represents the pooled HR and 95% CI.

**Figure 6:**
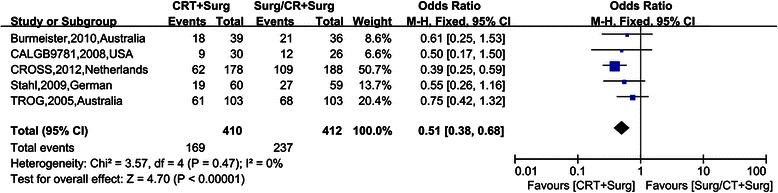
Forest plots for the secondary outcome postoperative recurrence rate. The squares and horizontal lines correspond to the study-specific HR and 95% CIs. The area of the squares reflects the study-specific weight. The diamond represents the pooled HR and 95% CI.

Figure 4, 5, 6, 7 show Forest plots for the secondary outcomes, including the R0 resection rate, positive rate of lymph node metastasis, postoperative recurrence rate, pathological complete response rate (pCR) and perioperative mortality. Five RCTs reported the R0 resection rate, indicating a statistically significant difference (OR 2.35, 95% CI 1.29–4.30, Figure 3). As the heterogeneity test was statistically significant (I^2^ = 59%), random effect modeling and subgroup analysis were performed, revealing a significant difference in the comparison between preoperative CRT and surgery alone (OR 3.55, 95% CI 2.34–5.39), while no statistically significant difference was observed in the comparison between preoperative CRT and preoperative CT (OR 1.17, 95% CI 0.61–2.27). Five RCTs reported a postoperative recurrence rate that included local and distant failure, indicating a statistically significant difference (OR 0.51, 95% CI 0.38–0.68, Figure 2), and no statistical heterogeneity was detected (I^2^ = 0%). Five RCTs reported the positive rate of lymph node metastasis according to the postoperative pathological report. The pooled OR was 0.30 (95% CI 0.23-0.39), revealing a significant difference, and no statistical heterogeneity was detected (I^2^ = 49%). Five RCTs reported perioperative mortality, and there was no statistically significant difference between the two groups (OR 1.10, 95% CI 0.62–1.93, Figure 4) nor a statistical heterogeneity (I^2^ = 0%). Six RCTs reported pCR after chemoradiation therapy, while two trials revealed pCR after chemotherapy, and the result of pCR in the chemoradiation therapy group was 21.56% (Table 1).

**Figure 7:**
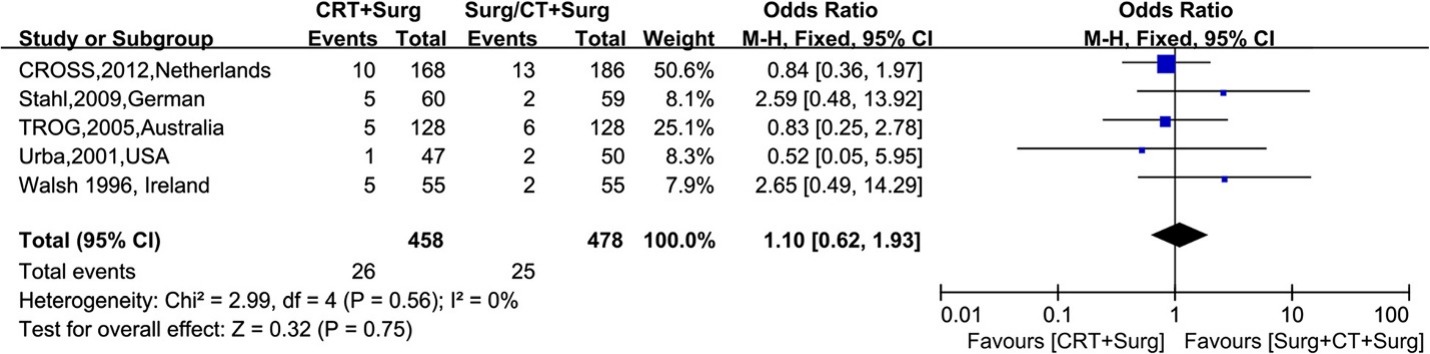
Forest plots for the secondary outcome perioperative mortality. The squares and horizontal lines correspond to the study-specific HR and 95% CIs. The area of the squares reflects the study-specific weight. The diamond represents the pooled HR and 95% CI.

## Discussion

Preoperative CRT has been used in the comprehensive treatment of GEJ and esophagus cancers for decades and has shown good curative effects in local control and prolonged overall survival. As early as 1978, Zhang had already carried out an elementary trial about preoperative CRT that confirmed that CRT was able to improve the results of surgery for GEJ cancer [34]. In the thirty years since, oncologists have put significant effort into the research of preoperative CRT and have had some success in the world scope. In the America, a phase II study proved that preoperative CRT was well tolerated and comparable to similarly staged, adjuvantly treated patients [35]. In Europe, a Spanish phase II study indicated that preoperative CRT showed an acceptable toxicity and promising activity [17], while a Polish phase II study revealed that CRT was effective and showed a good toxicity profile [18]. In Asia, a Japanese phase I study indicated that CRT might cause surgery to be delayed, but showed promise for resectable advanced gastric cancer, while a Korean phase I study showed that CRT could be explored more extensively [36]. Although all of these studies indicated a tendency for preoperative CRT to obtain more powerful evidence, this meta-analysis was conducted to evaluate preoperative CRT for patients with resectable esophago-gastric adenocarcinoma.

This meta-analysis is based on 7 RCTs published from 1996 to 2012. The most important achievement of this study is the result that patients with resectable esophago-gastric adenocarcinoma tended to have a survival advantage from preoperative CRT compared with surgery alone or preoperative CT followed by surgery. Although most of the individual HRs indicated no significant difference, the pooled HR revealed favorable results for the CRT group. The 3-year OS of Walsh’s study in the CRT and surgery group was 32% and 6%, while the median survival time was 16 months and 11 months (p < 0.01). Some individual data, such as the results above, indicated this opinion as well.

To identify the effect of adding radiotherapy to preoperative CT separately, we focused on the five RCTs that compared the survival benefit between preoperative CRT and surgery alone. The pooled HR was 0.75 (95% CI 0.62-0.90), which revealed a significant difference between these two groups. On the other hand, the results of the comparison between preoperative CRT and preoperative CT in the remaining two RCTs were disappointing because the pooled HR was 0.71 (95% CI 0.48-1.05), which compared with preoperative CT meant that patients may receive a benefit from preoperative CRT, but the effect was not significant. This result was consistent with the conclusion of another meta-analysis published previously [20]. We arrived at the deduction that preoperative CRT as a whole could bring a survival advantage for patients with esophago-gastric adenocarcinoma; however, limited to the number of RCTs that compared the effect between preoperative CRT and CT, we were not able to confirm the effect of radiotherapy separately. Perhaps there was a potential difference between preoperative CRT and CT; however, this difference was not observed due to the restricted number of RCTs. Therefore, the true benefit of radiotherapy separately might be much greater, and efforts to enlarge the simple size to prove the supposition are warranted.

To determine the reason that patients with resectable esophago-gastric adenocarcinoma could receive a survival advantage from preoperative CRT, we chose the R0 resection rate, positive rate of lymph node metastasis, postoperative recurrence rate and pathological complete response rate as secondary outcomes. The final results revealed that the pooled ORs of the R0 resection rate, positive rate of lymph node metastasis and postoperative recurrence rate favored the group of preoperative CRT. Furthermore, the combined pCR rate of six RCTs was 21.56%, which approximated the results of other studies [19,37,38]. According to the data above, downstaging as a result of preoperative CRT was reflected in the significantly higher percentage of the negative rate of lymph node metastasis and pCR rate. Therefore, our analyses concluded that downstaging, the possibility of complete resection and the decreased likelihood of local recurrence as a local control rate, which were the mechanisms of preoperative CRT, prolong survival. In addition, the pooled ORs of the R0 resection rate (OR 1.17, 95% CI 0.61-2.27) and postoperative recurrence rate (OR 0.57, 95% CI 0.32-1.02) were both non-significant, while only the pooled OR of the positive rate of lymph node metastasis (OR 0.40, 95% CI 0.22-0.72) was significant. This result also proved the conclusion above that compared with preoperative CT group; the local control rate responsible for the survival benefit was provided with a rising trend in the preoperative CRT group. However, further RCTs were necessary. Moreover, our analyses found that the pooled OR of perioperative mortality (OR 1.10, 95% CI 0.62-1.93) was non-significant, which meant that preoperative CRT was safe and tolerable.

There were some other valuable studies that compared the effects of preoperative CRT and surgery alone in patients with resectable esophago-gastric adenocarcinoma that were not included in this meta-analysis. The FFCD9901 trial focused on the survival outcomes for patients with localized (stages I or II) resectable esophageal carcinomas [39]; however, it is regrettable that the survival result was non-significant. We excluded this article because 75% of the patients suffered from squamous cell carcinoma and two thirds of the tumors were node-negative, which might be the cause of the negative results. Furthermore, a phase II study released on the 2013 ESMO meeting that took a therapeutic regimen as inducing chemotherapy followed by concurrent CRT before surgery reached a favorable result. The downstaging rate was 67%, while the pCR rate was 18%. This study mainly focused on the gastric adenocarcinoma and involved tumors on the antrum of the stomach. As its special value, a larger randomized trial is expected. From the clinical trial database, we also found an ongoing RCT from Australia that compared the survival differences between preoperative CRT and CT. Patients with adenocarcinoma of the stomach or gastroesophageal junction were included. It remains to be seen whether the final result will be favorable.

## Conclusion

All of the studies included in our meta-analysis are RCTs, and we were fortunate to reach a significant result with slight heterogeneity, which was resolved by subgroup analyses. Therefore, our meta-analysis demonstrates that patients with resectable esophago-gastric adenocarcinoma can gain a survival advantage from preoperative CRT. However, due to the limitations of the number of RCTs, the benefit of adding radiotherapy to preoperative CT separately is still uncertain and additional high-quality prospective trials are needed.

## Abbreviations

CRT: Preoperative chemoradiation therapy
pCR: Pathological complete response
HR: Hazard ratio
OR: Odds ratio
RCTs: Randomized controlled trials
CI: Confidence interval
CT: Preoperative chemotherapy
CENTRAL: Cochrane Central Register of Controlled Trials
EMBASE: Excerpta Medica Database
CNKI: China National Knowledge Infrastructure
ITT: Intention-to-treat
PRISMA: Preferred Reporting Items for Systematic Reviews and Meta-Analyses
OS: Overall survival
SCC: Squamous cell carcinoma
AD: Adenocarcinoma
